# Childhood Adversity and Dimensional Variations in Adult Sustained Attention

**DOI:** 10.3389/fpsyg.2020.00691

**Published:** 2020-04-16

**Authors:** Sarah C. Vogel, Michael Esterman, Joseph DeGutis, Jeremy B. Wilmer, Kerry J. Ressler, Laura T. Germine

**Affiliations:** ^1^Department of Applied Psychology, New York University, New York, NY, United States; ^2^National Center for PTSD, VA Boston Healthcare System, Boston, MA, United States; ^3^Boston Attention and Learning Laboratory, VA Boston Healthcare System, Boston, MA, United States; ^4^Department of Psychiatry, Boston University School of Medicine, Boston, MA, United States; ^5^Department of Psychiatry, Harvard Medical School, Boston, MA, United States; ^6^Department of Psychology, Wellesley College, Wellesley, MA, United States; ^7^Institute for Technology in Psychiatry, McLean Hospital, Belmont, MA, United States; ^8^Division of Depression and Anxiety Disorders, McLean Hospital, Belmont, MA, United States

**Keywords:** sustained attention, childhood adversity, psychopathology, cognition, population-based

## Abstract

**Background and Objective:**

Sustained attention is a transdiagnostic phenotype linked with most forms of psychopathology. We sought to understand factors that influence the development of sustained attention, by looking at the relationship between childhood adversity and adult sustained attention.

**Participants, Setting, and Methods:**

Participants were 5,973 TestMyBrain.org visitors from English-speaking countries who completed a continuous performance task (gradCPT) of sustained attention and a childhood adversity questionnaire. We analyzed gradCPT performance using a signal detection approach.

**Results:**

Discrimination ability (the main metric of performance on the gradCPT) was associated with total childhood adversity load, even when controlling for covariates related to age, gender, parental education, race, country of origin, and relative socioeconomic status (β = −0.079, *b* = −0.032).

**Conclusion:**

Our results demonstrate that attention differences related to childhood adversity exposure can (1) be measured using brief, performance-based measures of sustained attention, (2) persist into adulthood, and (3) be detected at the population level. These results, paired with the well-documented associations between sustained attention and psychopathology, indicate that sustained attention may be an important mechanism for understanding early influences on mental health.

## Introduction

Attention is a vital part of daily functioning, and the ability to sustain attention to a stimulus or task for an extended period of time has implications for employment (Biederman and Faraone, 2006), education (Frazier et al., 2007), vehicle safety (Barkley et al., 1996), and adaptive daily functioning (Gjervan et al., 2012). Sustained attention impairment is a feature of almost every major psychiatric illness (Liu et al., 2002; Politis et al., 2004; Avisar and Shalev, 2011). Sustained attention deficits are consistently identified in schizophrenia (Liu et al., 2002; Politis et al., 2004), bipolar disorder (Liu et al., 2002; Clark et al., 2005), anxiety (Ballard, 1996; Forster et al., 2015), PTSD (Heinrichs, 2001; DeGutis et al., 2015), and major depression (Weiland-Fiedler et al., 2003; Politis et al., 2004).

Most studies examining the effects of childhood adversity on sustained attention have focused on cognitive abilities in children, or on clinical deficits in attention associated with ADHD. Loman et al. (2013) studied a sample of internationally adopted post-institutionalized (PI) 10- and 11-year olds. PIs exhibited poorer performance on tests of sustained attention compared to non-adopted children. Several studies have identified both genetic and environmental factors in the development of ADHD (Knopik et al., 2005; Larsson et al., 2013), with childhood adversity being particularly related to development of ADHD in children (Biederman et al., 1995; Banerjee and Duflo, 2007; Froehlich et al., 2011).

Most literature has found mixed or no evidence of persistent effects of childhood adversity on adult attention. Lara et al. (2009) surveyed an international sample of adults for self-reported symptoms of ADHD and childhood adversity exposure. Childhood ADHD was significantly related to exposure to most adversities measured, while adult ADHD was only associated with parental psychopathology. Kessler et al. (2005) had similar findings in a retrospective self-report study of childhood ADHD and current ADHD in a sample of 3,197 adults. Childhood adversity was significantly related to childhood ADHD, but not adult ADHD. These findings point to the need for more research to understand the effects of childhood adversity exposure on variations in adult sustained attention outside of clinically significant attention deficits.

Studies using *objective performance-based measures* to understand the effects of childhood adversity on adult sustained attention are limited by small sample size and have had mixed results. In a sample of 47 adults, Majer et al. (2010) found no association between childhood trauma and adult sustained attention. In a sample of 66 veterans, Fortenbaugh et al. (2017) found significant impairments in sustained attention, assessed using the Gradual Onset Continuous Performance Test (gradCPT), in those with interpersonal early life trauma exposure. These individuals showed increased functional connectivity between the amygdala and prefrontal cortex and decreased functional connectivity between the amygdala and parahippocampal gyrus compared to those without childhood adversity exposure. Similarly, Lim et al. (2016) found that adult participants (*n* = 70) with a history of child abuse performed worse on an objective sustained attention task and showed decreased activation in the left inferior and dorsolateral prefrontal cortex, areas of the brain associated with sustained attention. Altogether, the literature suggests that deficits in sustained attention attributable to childhood adversity may persist into adulthood, but that such effect sizes are modest and may rely on the inclusion of sensitive measures of sustained attention in adequately powered samples.

Given shifts in the conceptualization of mental disorders as dimensional variations in neurobiology and observable behavior (RDoC; Cuthbert and Insel, 2013), understanding how adversity might contribute to individual differences in sustained attention and the persistence of these differences is a critical step in determining how mental illness might be related to sustained attention. The aim of the current study is to determine whether childhood adversity exposure is associated with variations in adult sustained attention in a well-powered, population-based sample. We used a reliable and sensitive measure of sustained attention (gradCPT; Esterman et al., 2013, 2015; DeGutis et al., 2015; Fortenbaugh et al., 2015; Riley et al., 2016) that has been validated across the lifespan and for remote web-based assessment (Fortenbaugh et al., 2015).

We relied on a large population-based design, including 1000s of subjects globally, to understand whether childhood adversity might account for dimensional variations in sustained attention outside of clinically ascertained samples. Such an approach permits broader generalizability and an initial estimate of population-level effect sizes and public health impact. We hypothesized that higher levels of childhood adversity exposure would be associated with poorer sustained attention performance, as measured by the gradCPT. Support for our hypothesis would provide a potential mechanism whereby adversity might be associated with transdiagnostic risk for psychopathology through impact on basic neural systems that support sustained attention.

## Materials and Methods

### Participants

Participants were visitors to www.TestMyBrain.org who completed a test battery called “Attention and Life Experiences” or “Concentration and Life Experiences” bearing the description “Test your concentration ability and help us understand how attention relates to life experiences. Of those who completed the experiment, we excluded those who: (1) indicated they had previously participated in this experiment or a similar one (6.61%), (2) reported having technical problems that may have interfered with their ability to complete the task or respond (2.67%), (3) reported any gender other than male or female (0.33%), (4) reported an age under 18 or over 65 (10.19%), or (5) opted out of the TestMyBrain Childhood Experiences Questionnaire (12.4%). Our final analytic sample comprised 5,973 visitors (40.57% female, mean_age_ = 36.73, *SD*_age_ = 12.72) to TestMyBrain.org from English-speaking countries: the United States, the United Kingdom, Ireland, Australia, Canada, and New Zealand. Sociodemographic information about our sample is included in Table 1 below.

**TABLE 1:** Demographic characteristics and adversity exposure rates in our analytic sample.

Number of participants	*n* = 5973		
**Gender**	***n*(percentage)**	**Adversity exposures**	***n*(percentage)**

Male	3526(59.03)	Verbal abuse	1871(31.32)
Female	2423(40.57)	Parental mental illness	1559(26.10)
No response	24(0.4)	Parent divorce	1555(26.03)
		Fear of abuse	1380(23.10)
**Race/ethnicity**		Parent alcoholism	1097(18.37)
Black/African	125(2.09)	Physical abuse: kick/hit	986(16.51)
Asian	791(13.24)	Neglect: supervision	978(16.37)
White/European	4484(75.07)	Physical abuse: push/grab	862(14.43)
Other	136(2.28)	Sexual abuse: touching	733(12.27)
Two or more races	292(4.89)	Domestic violence: push/grab	707(11.84)
No response/decline to respond	145(2.43)	Dangerous chores	431(7.22)
Hispanic/Latino	309(5.17)	Domestic violence: kick/hit	391(6.55)
****		Physical abuse resulting in injury	374(6.26)
**Participant education level**		Parent death	355(5.94)
Middle school	17(0.28)	Neglect: clothing	295(4.94)
High school	317(5.31)	Sexual abuse: intercourse	289(4.84)
Some college	1186(19.86)	Parental imprisonment	288(4.82)
College	1899(31.79)	Hunger due to poverty	281(4.70)
Graduate school	2114(35.39)	Neglect: medical	272(4.55)
None of the above	331(5.54)	Parental suicide attempt	246(4.12)
Decline to respond/no response	109(1.82)	Neglect: hunger	242(4.05)
**Parental education level**		Parental drug abuse	195(3.26)
Less than high school	428(7.17)	Foster care	113(1.89)
High school diploma	1633(27.34)	Institutional care	110(1.84)
College	1630(27.30)	Parent criminal activity	83(1.40)
Master’s degree	1031(17.26)		
Doctorate or equivalent	1042(17.45)		
Don’t know/decline to respond	209(3.50)		
**Relative SES**			
Much lower	322(5.39)		
Lower	1017(17.03)		
About the same	2241(37.52)		
Higher	1832(30.67)		
Much higher	459(7.68)		
Don’t know/decline to respond	102(1.71)		

TestMyBrain.org is a not-for-profit research initiative that uses a citizen science model to engage everyday people in research studies. People participate in studies on TestMyBrain in exchange for feedback on their scores. No explicit advertising or recruitment is conducted. Participants come to TestMyBrain from search engines or through previous participants. The quality of cognitive data collected on TestMyBrain.org is comparable to data collected using traditional methods (Germine et al., 2012) including for this specific task (Fortenbaugh et al., 2015), and produces convergent results with studies in other population-based samples (Halberda et al., 2012; Hartshorne and Germine, 2015).

### Measures

#### Sustained Attention

**FIGURE 1:**
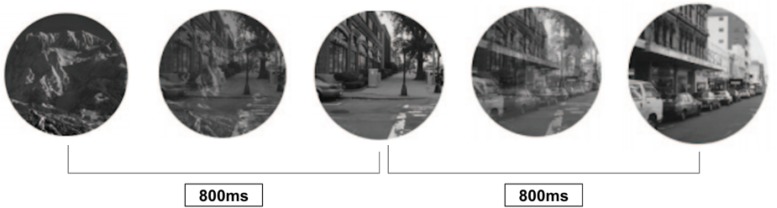
Illustration of gradCPT procedures, adapted from Fortenbaugh et al. (2015).

The gradCPT is a measure of sustained attention and response inhibition that was developed and validated as an individual differences measure for investigations of brain and behavior (Esterman et al., 2013, 2015; DeGutis et al., 2015; Fortenbaugh et al., 2015; Riley et al., 2016). The test includes 10 grayscale photographs of city scenes and 10 grayscale mountain scenes. Participants were instructed to press a button whenever a city scene appeared, and do nothing when a mountain scene appeared. The photos were presented in fixed random order over 300 trials, with 10% mountain scenes and 90% city scenes and no duplicate scenes appearing consecutively. The photos transitioned pixel-by-pixel over 800 ms to reduce alerting effects of a discrete inter-trial interval, increasing difficulty and attentional fatigue (Esterman et al., 2013, 2015). Reaction times (RTs) were calculated relative to the beginning of the transition between images. See Figure 1 for an illustration of the gradCPT procedure (adapted from Fortenbaugh et al., 2015). Performance on the gradCPT has shown to be lower in patient populations that traditionally exhibit attention problems (DeGutis et al., 2015), and correlates with self-reported attention problems in everyday life (Rosenberg et al., 2013). The potential distractors associated with self-administration outside the laboratory present an ecologically valid method of assessing sustained attention ability in the real world, where such distractions are almost always present (Rosenberg et al., 2013).

#### Childhood Adversity

We used the TestMyBrain Childhood Experiences Questionnaire to assess childhood adversity experiences from birth until 18. This questionnaire was adapted from the Adverse Childhood Experiences Scale, Conflict Tactics Scale, and Composite International Diagnostic Interview and has been used previously to examine the impact of childhood adversity on social cognition at the population level (Germine et al., 2015). Participants were asked whether they had been exposed to the following experiences (listed in Table 1 above): parental death, parental divorce, institutionalization, foster care, parental alcoholism, parental drug abuse, parental mental illness, parental suicide, parental imprisonment, and sexual abuse. The answer options were “yes,” “no,” “I don’t know,” or “I’d rather not say.” Participants also reported on frequency of the following adversities: parental criminal behavior, domestic violence, verbal abuse, physical abuse, fear of abuse, family dependence on welfare assistance, hunger due to poverty, exposure to dangerous chores, and parental neglect. The answer options were “often,” “sometimes,” “rarely,” and “never.” The survey can be viewed at https://testmybrain.org/tests/childhood_adversity.html.

### Procedure

Participants reporting an age of 18 and over completed the gradCPT, followed by the childhood adversity questionnaire and a basic demographic questionnaire at the end. The gradCPT consists of instructions, practice, and a 4-min assessment. Participants were alerted to the sensitive material in the childhood adversity questionnaire and could opt out and complete a different questionnaire if they desired. All procedures were approved by the Harvard University Committee on the Use of Human Subjects in Research (CUHS).

### Analyses

#### Total Adversity Score

Our primary analysis of childhood adversity used a total adversity score as the sum of all the exposure scores (similar to Friedman et al., 2015), which allowed for a single omnibus test of the hypothesis that childhood adversity is associated with poorer sustained attention in adulthood. For purposes of analysis, adversity exposures were recoded as “exposed” or “not exposed.” Participants answering “yes,” “sometimes,” or “often” were coded as “exposed” (coded as 1). Participants answering “no,” “rarely,” or “never” were coded as “not exposed” (coded as 0). We chose to recode into “exposed” and “not exposed” groups to account for the different answer options for questions throughout the assessment and facilitate comparisons across questions. A total adversity score was then calculated as the sum of all adversity exposures. Importantly, the childhood adversity questionnaire asked about family reliance on government assistance (welfare), however due to large variations in availability and use of government assistance across countries, such information was excluded from this analysis. Thus, each participant had a total adversity score that varied between 0 (no exposure) and 25 (exposure to all adversities included in the survey). This coding scheme is similar to the coding scheme for the Adverse Child Experiences Questionnaire (ACE) (Felitti et al., 1998).

#### Sustained Attention Performance

We analyzed gradCPT data using a signal detection theory approach (Fortenbaugh et al., 2015). Errors on gradCPT trials were classified as either commission (failure to inhibit a response to a mountain scene) or omission errors (failure to respond to a city scene). We then computed d′ (discrimination ability) and criterion (willingness to respond in cases of uncertainty) based on hit rate (percentage of correct response omissions to mountains) and false alarm rate (percentage incorrect omissions to city scenes). We scaled and centered commission and omission rate values to use in calculating the signal detection measures. The hit rate was calculated by subtracting the commission error rate from 1, and the false alarm rate was just the omission error rate. This method of calculating hits and false alarms is standard procedure for the gradCPT (see Fortenbaugh et al., 2017; Rothlein et al., 2018 for examples). The target action in the gradCPT is *inhibition of a response*, rather than a response itself. Thus, in this task, a “hit” is considered a correct inhibition of a response (doing nothing in response to a mountain scene), while a “false alarm” is considered an incorrect inhibition of a response (failure to respond to a city scene). Using these metrics we then calculated d′ as the difference between the hit rate and the false alarm rate. To calculate the criterion, we took the sum of the hit rate and the false alarm rate and divided by two, then multiplied by negative one. To correct for 100% hit rate or 0% false alarm rate we used standard procedures and added or deducted 0.001 to omission or commission rate in such cases. d′ represents the participant’s ability to discern between a mountain scene and a city scene. Criterion is a metric of strategy, or willingness of a participant to respond when they are unsure if the scene is a city or a mountain.

For correct trials, we also calculated mean RT and coefficient of variability for each participant (CV; the standard deviation of RT divided by mean RT; Fortenbaugh et al., 2015). We used multiple linear regression to test the hypothesis that variations in childhood adversity load are related to individual differences in adult sustained attention performance, with adversity load as a predictor of each of four sustained attention measures derived from the gradCPT.

#### Covariates

Covariates included linear and quadratic effects of age, gender, relative SES, participant race/ethnicity, highest parental education level (whichever was higher between paternal and maternal education level), and country of origin. Relative SES is the participant’s self-report of their socioeconomic status relative to the average in the community they grew up in (much lower, lower, middle, higher, much higher). Raw and standardized effect sizes are reported along with 95% confidence intervals (CIs).

## Results

### Exposure to Childhood Adversity

**FIGURE 2:**
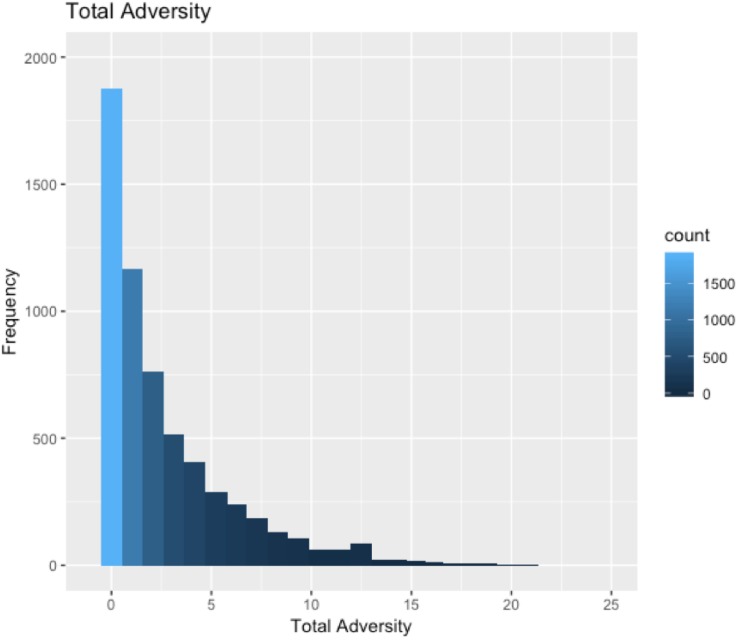
Histogram of total adversity scores in this sample.

A histogram of total adversity scores is shown in Figure 2 (mean = 2.63, median = 1, *SD* = 3.26, range: 0–21). Rates of individual adversity exposures in our sample are shown in Table 1. 68.56% of participants in our sample reported being exposed to at least one form of adversity, rates of individual adversity exposures are shown in Figure 2. Total adversity scores were higher among females than males [*t*(4650) = −8.852, *p* < 0.001], among participants with lower SES backgrounds [*r*(5969) = −0.277, *p* < 0.001], and among non-white participants [*t*(2248.5) = 3.434, *p* < 0.001].

### Differences in Discrimination Ability Related to Childhood Adversity

**FIGURE 3:**
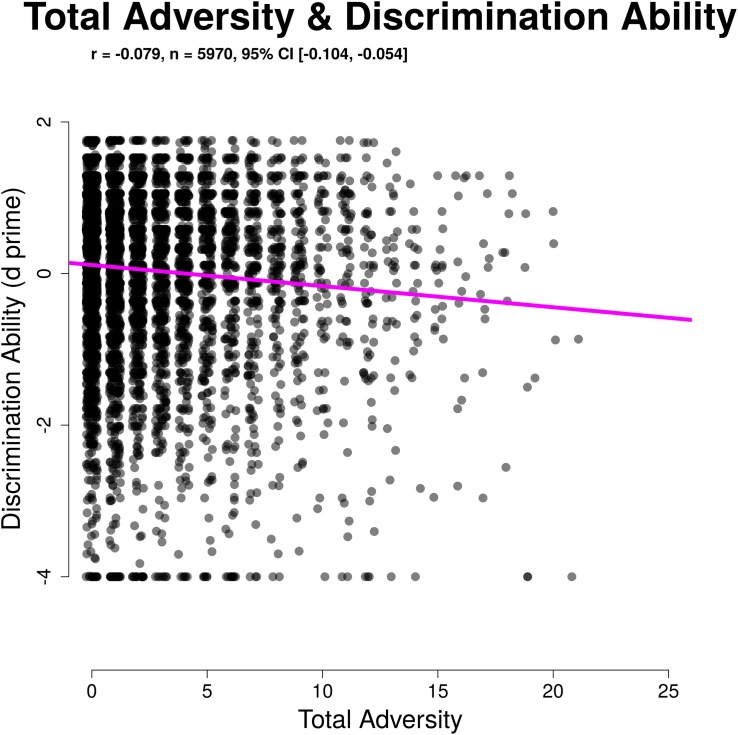
Significant association between total adversity and discrimination ability.

**FIGURE 4:**
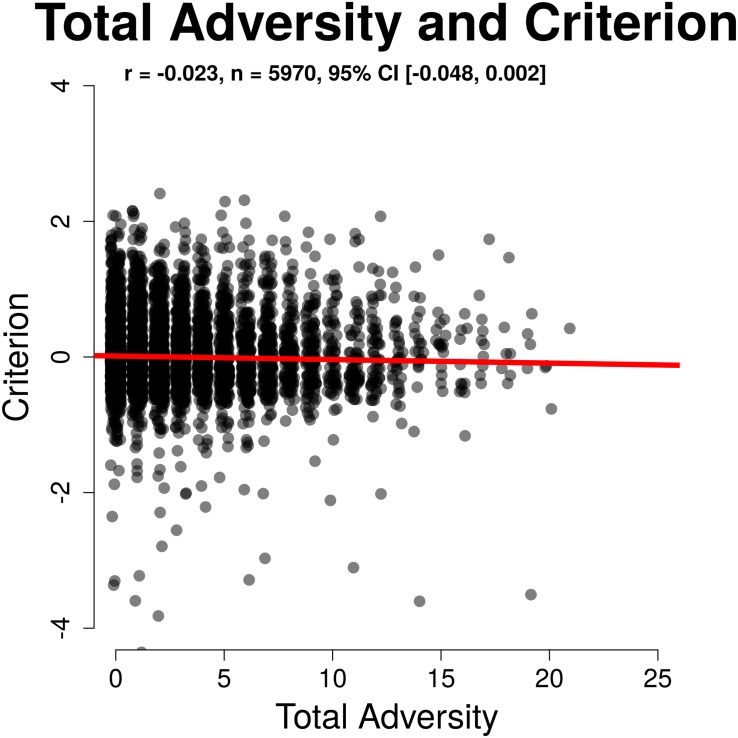
Non-significant relation between total adversity and criterion.

Discrimination ability (d′) was significantly related to total childhood adversity (*r*(5968) = −0.080, 95% CI [−0.105, −0.055], *p* < 0.001). This relationship remained after controlling covariates described above (β = −0.079, 95% CI [−0.090, −0.068], *b* = −0.032, *p* < 0.001), with greater adversity load associated with poorer performance (lower d′). Response criterion had a trend level relationship with total childhood adversity (*r*(5968) = −0.023, 95% CI [−0.048, 0.002], *p* = 0.074) meaning that although greater adversity load was somewhat related to greater willingness to respond, this relationship was not significant, nor was it robust to covariates. Overall, our results indicate that childhood adversity exposure is related to poorer sustained attention with minimal differences in task approach or strategy after accounting for sociodemographic differences that vary with adversity exposure. These relationships are illustrated in Figures 3, 4.

### Differences in Reaction Time Related to Childhood Adversity

**FIGURE 5:**
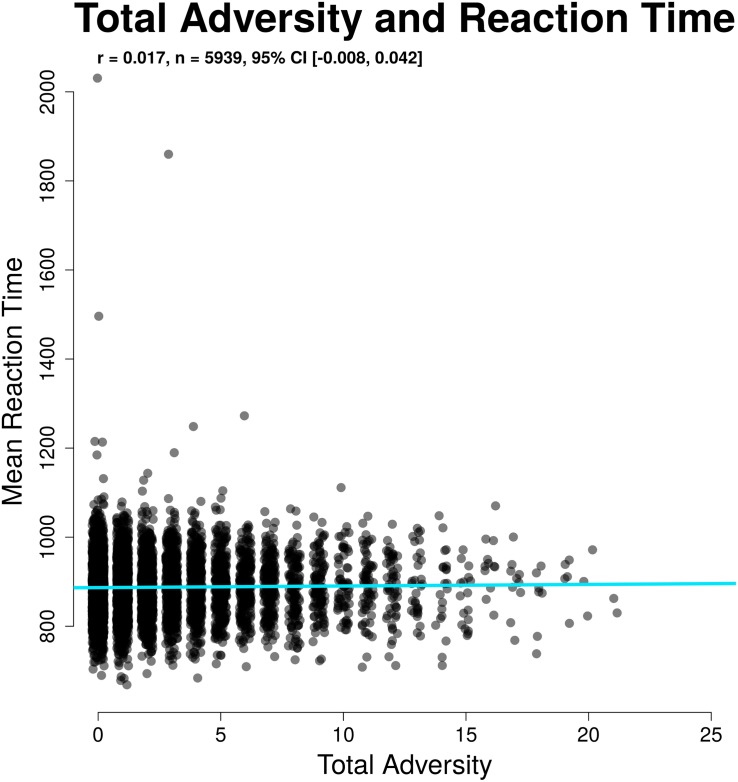
Non-significant relation between adversity and mean RT on correct trials.

**FIGURE 6:**
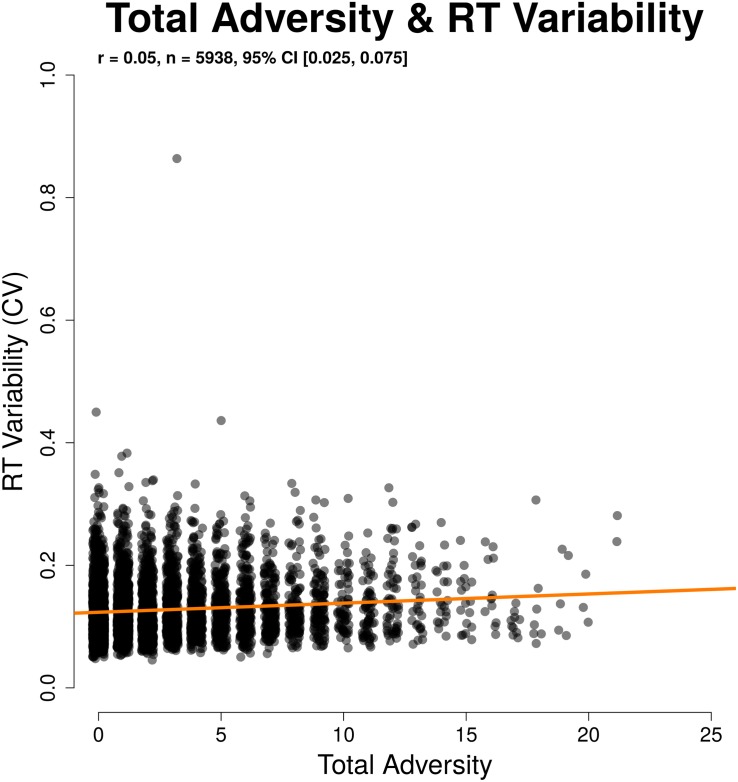
Significant relation between adversity and reaction time (RT) variability, as measured using a coefficient of variability.

To understand the relationship between RT and adversity, we examined mean RT and RT variability (CV) on correct city trials. Mean RT was not related to total adversity (*r*(5937) = 0.017, 95% CI [−0.009,0.042], *p* = 0.2). CV was higher among participants with higher total adversity load (*r*(5936) = 0.050, 95% CI [0.025,0.076], *p* < 0.001). This relationship for CV held when controlling for age, gender, parental education, and SES (β = 0.0746, 95% CI [0.0742, 0.0750], *b* = 0.001, *p* < 0.001). These results suggest that adversity might impact the stability of task performance, even where overall speed of responses is unaffected. The results here mirror the results for d′ and criterion, as d′ is more closely associated with CV and criterion with mean RT on this task (see Fortenbaugh et al., 2015). These relationships are illustrated in Figures 5, 6.

Summaries of the estimates effect sizes and CIs for all findings are presented in Table 2 above.

**TABLE 2:** Effect sizes and confidence intervals for all reported regressions and correlations.

Predictor	Outcome	Estimated effect size	95% Confidence interval
Total adversity	Discrimination ability (d′)	β = −0.079	[−0.090, −0.068]
Total adversity	Criterion	*r*(5968) = −0.023	[−0.048, 0.002]
Total adversity	Mean RT	*r*(5937) = 0.017	[−0.009, 0.042]
Total adversity	CV (RT variability)	β = 0.0746	[0.0742, 0.0750]

## Discussion

In this study we used a large, population-based sample to assess the impact of childhood adversity exposure on adult sustained attention. Our results suggest that higher levels of childhood adversity are associated with poorer sustained attention in adulthood. Specifically, increases in adversity load were associated with linear decreases in discrimination ability (d′) and increases in RT variability (CV). These associations remained after controlling for sociodemographic factors, confirming that these effects could not be attributed to gender, age, or socioeconomic differences that might vary with childhood adversity exposure and/or sustained attention.

While the effect sizes reported here are quite small, they are robust to a number of demographic covariates, and provide an estimate of the degree of population-level variation in sustained attention ability that can be attributed to childhood adversity load. Previous studies in this area have relied on small, clinical samples that were not powered to detect small effect sizes, or on large samples focused on self-reported clinically significant attention deficits. One of the main strengths of this study was the large sample size and sensitive performance-based measure of attention, which allows us to detect even small differences in sustained attention ability at a population level. Further research is needed to investigate the extent to which small variations in sustained attention ability pose a meaningful difference to daily functioning. This study provides a much-needed foundational step toward such an investigation.

Here we relied on a well-validated, sensitive, and reliable test of sustained attention (Esterman et al., 2013, 2015; DeGutis et al., 2015; Fortenbaugh et al., 2015; Riley et al., 2016), administered to a large and diverse adult sample, allowing us to overcome potential limitations of previous studies. Previous research has indicated that childhood adversity is associated with poorer attention in childhood (Kessler et al., 2005; Lara et al., 2009; Loman et al., 2013), with most findings centered on ADHD (Biederman et al., 1995; Kessler et al., 2005; Banerjee and Duflo, 2007; Lara et al., 2009; Froehlich et al., 2011). Our results extend these findings and show a negative association between childhood adversity and sustained attention in adults. A population-based approach that focuses on individual differences is consistent with the RDoC framework of understanding dimensional variations in fundamental domains of information processing (Cuthbert and Insel, 2013). Previous studies examining adult sustained attention and childhood adversity exposure relied on small samples or used relatively insensitive measures that are not validated for measuring individual differences (Majer et al., 2010; Fortenbaugh et al., 2017).

Traditionally, research on cognition and adversity relies on case-control studies where cases are recruited from groups with extreme forms of adversity (Nelson, 2007; Hostinar et al., 2012; Loman et al., 2013; Roeber et al., 2014) or selected based on clinical characteristics. These studies have provided a foundational understanding of the way childhood adversity might impact the development of cognition, but do not provide information about how much childhood adversity experiences impact cognition at the population level. Our findings provide an initial estimate of how much adult variability in sustained attention may be attributable to early experiences of adversity.

There are several potential mechanisms that might explain the association between childhood adversity and adult sustained attention. Previous work has linked childhood maltreatment with differences in the anatomy and function of prefrontal regions (e.g., left inferior frontal gyrus) that have been linked with sustained attention and cognitive control (Lim et al., 2016). Fortenbaugh et al. (2017) found that individuals with interpersonal early life trauma experiences performed significantly worse on the gradCPT than healthy controls, and that these impairments where correlated with increased functional connectivity between the amygdala and prefrontal cortex. These results suggest that childhood adversity might impact the development of frontal cognitive control systems and their connections with subcortical affective processing systems, negatively impacting sustained attention and cognitive inhibition.

Our study relied on self-administered, web-based assessments of sustained attention using an objective performance-based task. It has been demonstrated that the gradCPT, assessed in this manner, has similar psychometric characteristics to traditional lab-based assessment of the same measure (Fortenbaugh et al., 2015). Several studies have now shown that performance on web-based cognitive measures is similar to performance in traditional in-person settings (Wilmer et al., 2010; Germine et al., 2012; Halberda et al., 2012; Hartshorne and Germine, 2015), including performance on the gradCPT (Fortenbaugh et al., 2015). Results on performance-based cognitive tests assessed across the lifespan also converge with findings in nationally representative samples (Hartshorne and Germine, 2015). We expect that web-based sampling methods likely impact the magnitude of estimated associations between childhood adversity and sustained attention (e.g., by oversampling from individuals with adversity exposure who self-select into the study), but nevertheless yield a starting point for understanding how childhood adversity and adult attention are linked at the population level that can be pursued using more traditional epidemiological study designs. A limitation of this method is the potential for self-selection biases in our sample, with individuals experiencing cognitive difficulties potentially seeking out testing to validate their experiences or people with more lifetime adversity experiences seeking out resources to understand their experiences better. Additionally, avoidance is a common symptom of PTSD (Foa et al., 1995) so it could be the case that individuals with adversity-related PTSD are underrepresented in our sample.

A more significant limitation is our study design’s reliance on retrospective self-report of childhood adversity. It is possible that adults with sustained attention difficulties were more likely to recall childhood adversity experiences than those without such difficulties. Studies that rely on prospective outcomes provide stronger evidence of associations, but have limitations related to participants’ willingness to report recent or current adversity experiences (Hardt and Rutter, 2004). As with any study relying on retrospective self-report, replication in a prospective design permits stronger conclusions about potential associations – the results reported here provide information that would be useful in designing such studies as potential future directions.

## Conclusion

In this study, we sought to examine the relationship between childhood adversity exposure and variations in adult sustained attention. Our results demonstrate that attention differences related to childhood adversity exposure can (1) be measured using brief, performance-based measures of sustained attention, (2) persist into adulthood, and (3) be detected at the population level. Our results suggest that differences in sustained attention that are linked with childhood adversity might provide a mechanism linking adversity exposure with risk for psychopathology in adulthood. Within the RDoC framework (Cuthbert and Insel, 2013), understanding the factors that might shape fundamental processes like sustained attention allows us to form hypotheses about the way variations in such processes might impact public health.

## Data Availability Statement

The datasets generated for this study are available on request to the corresponding author.

## Ethics Statement

The studies involving human participants were reviewed and approved by the Harvard University Committee on the use of Human Subjects in Research. The patients/participants provided their written informed consent to participate in this study.

## Author Contributions

SV contributed analysis and writing to this manuscript. ME and JD provided guidance on study design and analysis, and writing feedback. JW provided guidance on data visualizations and writing assistance. KR provided guidance on study design and writing feedback. LG contributed to study design, analysis, and writing.

## Conflict of Interest

The authors declare that the research was conducted in the absence of any commercial or financial relationships that could be construed as a potential conflict of interest.
